# Successful Treatment of Holmes Tremor With Deep Brain Stimulation of the Prelemniscal Radiations

**DOI:** 10.3389/fsurg.2018.00021

**Published:** 2018-05-31

**Authors:** Vicente Martinez, Shu-Ching Hu, Thomas J. Foutz, Andew Ko

**Affiliations:** ^1^Department of Rehabilitation Medicine, University of Washington, Seattle, WA, United States; ^2^Department of Neurology, University of Washington, Seattle, WA, United States; ^3^Department of Neurosurgery, University of Washington, Seattle, WA, United States

**Keywords:** deep brain stimulation, DBS, holmes tremor, prelemniscal area, tractography

## Abstract

Holmes tremor (HT) is a rare movement disorder that is typically associated with cerebellar, thalamic or brainstem lesions following a delay. Treatment of HT with deep brain stimulation (DBS) has yielded positive results however; it is unclear which deep brain targets provide optimal therapeutic effects. Here we describe a case report in which a 34 year old man with HT treated successfully with DBS. The ventrointermediate nucleus (VIM) of the thalamus was considered as the initial target. Following electrode placement we determined that the ventral-most electrode contacts were located in the prelemniscal radiations (Raprl). When stimulating from the Raprl contacts, the patient demonstrated robust, stable therapeutic improvements using remarkably low voltages. Our case report corroborates prior evidence suggesting the Raprl as a viable therapeutic target for treating HT with DBS.

## Introduction

Holmes tremor (HT) has been described as a rare, low-frequency tremor (below 4.5 Hz) that includes resting, kinetic and postural components; HT is typically accompanied by ataxia, bradykinesia and ophthalmoplegia (1). The causes of HT may involve neurological insults that include traumatic brain injury, ischemic stroke, tumors, venous malformations, or neurological surgery. After an initial neurological insult, the onset of HT follows a delay of 1 to 24 months at which time tremor begins to manifest (2). Although sometimes referred to as “rubral tremor”, the causes are not limited to lesions of the red nucleus. Rather, lesions of the upper brainstem, thalamus, and cerebellum will produce this type of tremor by interrupting the cerebellar outflow tract (3–5). Here we report the case of a patient with bilateral thalamic lesions who developed HT over the course of 3–4 months and was responsive to DBS therapy in the prelemniscal radiations (Raprl).

The pharmacological treatment of HT commonly involves the simultaneous administration of medications for Parkinson disease (PD) or for essential tremor (ET), such as dopaminergic agents, anticholinergics, propranolol and primidone, however in most cases these medications are ineffective or intolerable (6). Evidence indicates that DBS therapy may provide tremor relief for patients with medically refractory HT. The preferred targets for treating HT with DBS include the ventral intermediate nucleus of the thalamus (VIM), the subthalamic nucleus (STN), and the globus pallidus interna (GPi) (7, 8). In some cases, successful therapy has been achieved by targeting multiple ipsilateral brain nuclei simultaneously (9). For example, Romanelli et al. (8) implanted an ipsilateral STN electrode in a patient for whom VIM stimulation improved intention and postural tremor, but failed to improve resting tremor; simultaneous VIM and STN stimulation successfully produced attenuation of all three tremor components whereas individual stimulation of these nuclei was less successful. Based on these findings, the authors posited that the mixed tremors observed in HT patients may be the result of the combined imbalance of cerebellothalamic and pallidothalamic pathways, and that successful treatment may necessitate the implantation of multiple DBS leads. In contrast to this multi-electrode approach, Toda et al. 2017 (10) utilized a single electrode to simultaneously stimulate VIM and the sub-thalamic area using an interleaved configuration; the effectiveness of this single-electrode/multi-target strategy lead the authors to suggest that a larger area of stimulation was required to treat HT than other tremor disorders. The success of multi-target DBS remains variable however, Lim et al. (11) described a HT patient implanted with VIM, VOA (ventralis oralis anterior) and GPI electrodes. Stimulation of the GPi alone was sufficient to produce tremor suppression, however the addition of thalamic nuclei stimulation failed confer any additional benefits. Collectively, these reports demonstrate that the efficacy of single or multi-target DBS to treat Holmes tremor appears to vary between patients. In addition to the use of multiple electrodes, the stimulation parameters required to produce therapeutic results using single-electrode configurations in HT (increased amplitude, pulse width and frequency, interleaved settings or multiple electrodes) typically exceed those required to treat PD or ET. This higher energy demand can shorten battery life and necessitate more frequent pulse-generators replacement surgeries. In this report, we present an alternative target for DBS within the prelemniscal radiations (Raprl) of a patient with longstanding, treatment-resistant HT. The patient’s severe right-upper extremity tremor was successfully treated using unilateral neurostimulation of the left prelemniscal area. Importantly, neurostimulation produced persistent and robust tremor control at the lowest amplitudes currently reported in the literature. The effectiveness of this approach corroborates existing evidence for Raprl as an effective, alternative neurosurgical target for the treatment of Holmes tremor with DBS.

## Case Report

A 45-year-old right-handed male initially presented to the University of Washington Movement Disorders Clinic in March of 2015. Informed written consent was obtained from the patient for the purpose of video-taping and case study publication. He attributed his HT to a fall in 2010, although he also had HIV infections and HIV-related neurological issues such as progressive multifocal leukoencephalopathy and toxoplasmosis. Although the cause of his lesions is unclear, given the location of the lesions, their appearance and the patient’s history of HIV, we surmise that they are the possible result of HIV-related vasculopathy associated with CNS toxoplasmosis. His career was in construction, and the onset of his neurological symptoms was associated with a fall through a roof in 2010. His immediate neurological status post-fall remains unknown; however, he reported the onset of multiple subsequent neurological deficits over the following three to four months, including tremor, seizures, gait impairments, left spastic hemiparesis and dysarthria. Subsequent MRI indicated the presence of bilateral thalamic lesions with the left lesion being substantially larger than the right. He first noted tremors of the left ring-finger, which spread throughout his left arm, then gradually involved his right arm. His right arm tremor represented the most active component of is Holmes tremor and displayed components of resting, postural and kinetic tremors. In addition to arm tremor he demonstrated low frequency tremors of the neck and chin. Consistent with the asymmetry of his thalamic lesions, his tremor was more severe on the right-body compared to his left, whereas rigidity symptoms displayed similar severity in bilateral upper and lower extremities. His tremors were refractory to medications including levodopa, propranolol, gabapentin, topiramate and primidone. Although he found some tremor improvement from baclofen 80 mg per day, this was associated with an exacerbation of his seizures, and worsened his already limited gait ability. Prior to surgery, progression of his symptoms including HT had plateaued, and his thalamic lesions likewise remained stable on imaging (see attached video: https://doi.org/10.6084/m9.figshare.5708275.v1).

He then underwent DBS surgery. Neuroimaging included intraoperative stereotactic CT scans, which were fused with previous MR images. The surgeon drilled a standard burr hole and opened the dura. Neurophysiological recordings were performed with the Medtronic Lead Point system. Two microelectrodes were lowered towards the VIM; neurophysiological confirmation of the target was based on the presence of large-spiking neurons. While recording, the electrode was advanced over 4.5 mm, exiting thalamus between 4 and 4.5 mm. The test electrode was then replaced with a quadripolar Medtronic DBS electrode (model 3387). During subsequent intraoperative macrostimulation, the patient demonstrated immediate tremor reduction in his right-arm with improved speech and no paresthesia. The electrode was left in placed given the marked therapeutic effect. We determined the target electrode (contact 0) to be approximately 3.5 mm off target, with contacts 0 and 1 located within the Raprl, inferior to the caudal zona incerta (CZi), and contacts 2 and 3 in the thalamus (SeeFigure 1). The coordinates of contact 1 within the Raprl were [x, y, z] −11.5, –6 and −2 mm. The coordinates of contact 2 were −12.6, –3.7 and 1.1 mm. The target location for Vim based on AC/PC was −13.7, –6.2 and 0 mm. His AC/PC distance is 25.51 mm. The Raprl classically is defined with respect to AC/PC distance divided by 10; the locations are of the electrode in relation to the Raprl are consistent with those delineated in the Schaltenbrand Atlas.

**Figure 1:**
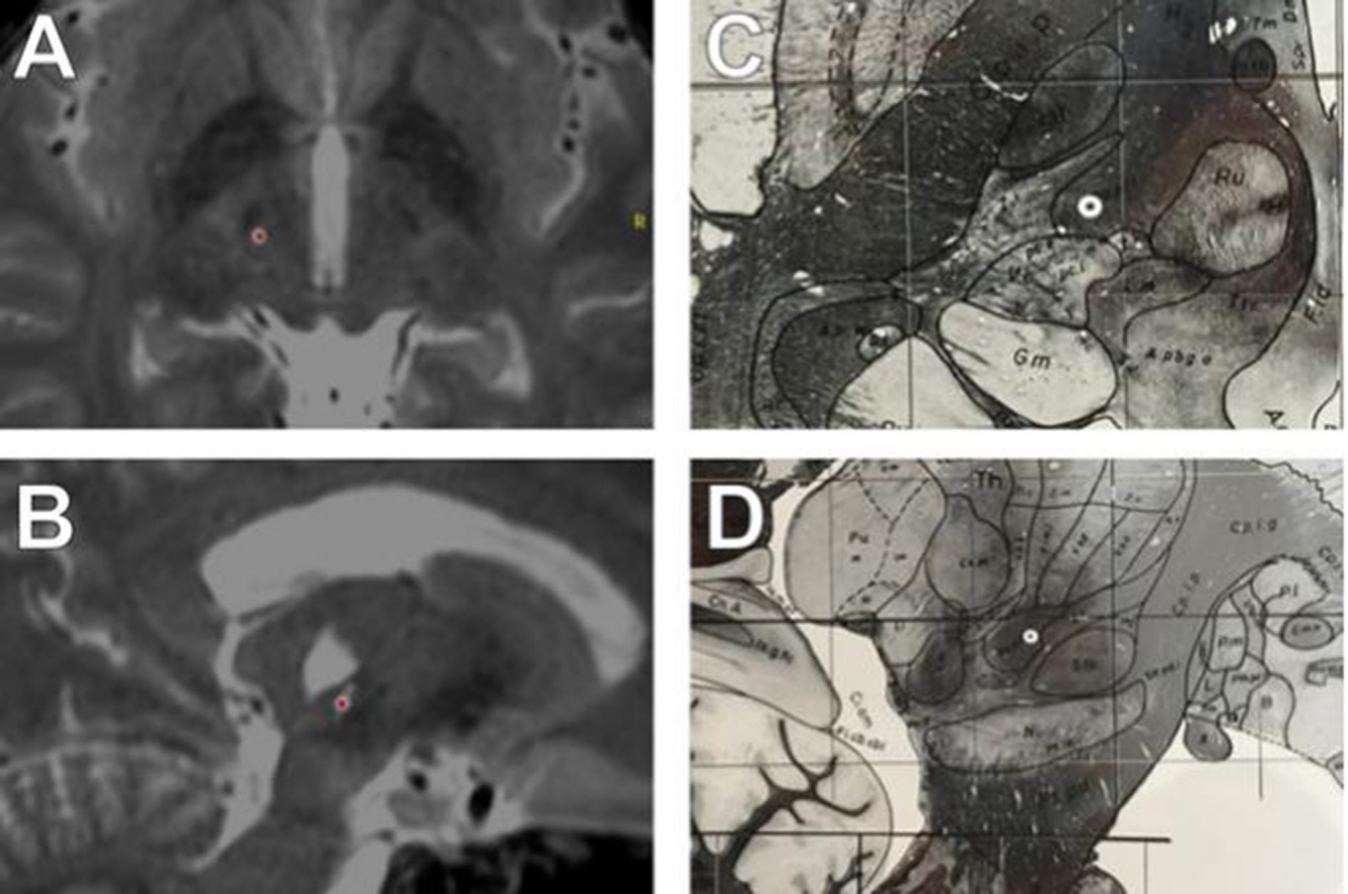
T2 MRI of electrode implantation site, axial **(A)** and sagittal **(B)** views. Target location on the Schaltenbrand and Wahren atlas (12) in axial **(C)** and saggital **(D)** views. Schaltenbrand G, Wahren W (12) Atlas for stereotaxy of the human brain. Stuttgart: Thieme. T2-SPACE MRI, merged with intraoperative CT imaging. **(A–B)**. The electrode is marked with a red dot. AC/PC coordinates were (Lat: −11, A-P: −5, Vert: −1.5). **(A)** Axial view, contact 1. Note STN is visible anterior to electrode location, with analogous relative location noted in C. **(B)** Sagittal view, contact 1. Note the electrode location on the sagittal view relative to STN in B, and D. Note also the large cystic lesion superior to the electrode within the thalamus. Electrode location, Schaltenbrand and Wahren atlas, with electrode location marked based on AC/PC coordinates (white circle). **(C)** Electrode location in axial plane (Plate 54, H.v −1.5). **(D)** Electrode location in the sagittal plane Schaltenbrand and Wahren, (Plate 44, S.l. 13)

It is likely that deviation from the intended trajectory was the result from the electrode encountering calcification or gliosis during advancement. The patient subsequently underwent implantation of a pulse generator (model 37612; Medtronic). He recovered well, and underwent programming approximately 4 weeks later. Monopolar mapping was performed with incremental voltage increases of 0.5 V (0–4.5 V), exploring all contacts. Contacts 0, 1 and 2 all produced marked therapeutic benefits at voltages below 3 V. However, the dorsal most contact (contact 3) failed to produce benefit at amplitudes up to 4.5 V. Based on these observations the selected configuration was with contact 2 negative, case positive, pulse width 90 microseconds, frequency 130 Hz, and voltage 2.7 V. The patient was instructed to resume their normal regimen of medications. Unfortunately, he suffered severe hand cramps, dizziness, and increased seizure frequency. He was seen for urgent reprogramming and stimulation was reduced to 2.0 V. Although this alleviated the patient’s cramping and reduced his seizure frequency, the lower setting produced insufficient tremor control and he had progressive worsening of his symptoms. On subsequent reprogramming, the more ventral contact 1 was assigned negative with case positive. This produced tremor control at remarkably low voltages. His selected configuration was with contact 1 negative, case positive, pulse width 90 microseconds, frequency 130 Hz and voltage 1.0 V. This resulted in a therapeutic impedance of 1,303 Ohms and a therapeutic current of 0.782 mA. At follow-up clinic evaluation, his tremor and rigidity were decreased substantially and the effects have been sustained for upwards of 6 months. Although his mobility is still limited, he has displayed improved gait. Furthermore, he is now able to write his name with a pen and has resumed previous hobbies of building models, making origami and airbrushing, none of which were possible prior to DBS surgery. His speech has demonstrated substantial improvement from his tendency to slur words, and he reports the quality of life is much improved. The beneficial effects of stimulation have been maintained and have not subsided for over 2 years.

The patient’s CT and MRI were co-registered via a standard linear registration algorithm. The modeled electrode was placed manually as close as possible to the center of the CT-defined artifact, which was confirmed on axial, sagittal and coronal MRI scans. After placement of the electrode, the stimulation parameters were input into the StimVision computer software, which generated an approximate volume of tissue activation using a validated artificial neural network predictor function (13–15). Model parameters were 1V stimulation with contact 1 negative, case positive, and pulse width 90 microseconds. Volumes were then used as seed region for fiber tractography. Tractography was performed using FSL (Analysis group, FMRIB, Oxford, UK). Preoperative diffusion-weighted images were acquired with reversed phase-encode blips, resulting in pairs of images with distortions going in opposite directions. The susceptibility-induced off-resonance field was estimated (16), eddy current distortions corrected, and local modelling of diffusion parameters was performed using default bedpost parameters (2 fibers/voxel) (17), with subsequent linear coregistration with anatomic MRI and postoperative CT imaging. Masks for the superior cerebellar peduncle and dentate gyrus were created manually, and along with the VTA estimation, were used as waypoint masks to generate the fiber model. Computed fibers travelled downward to the dentate nucleus of the cerebellum, and upwards towards the M1-and premotor areas (SeeFigure 2).

**Figure 2:**
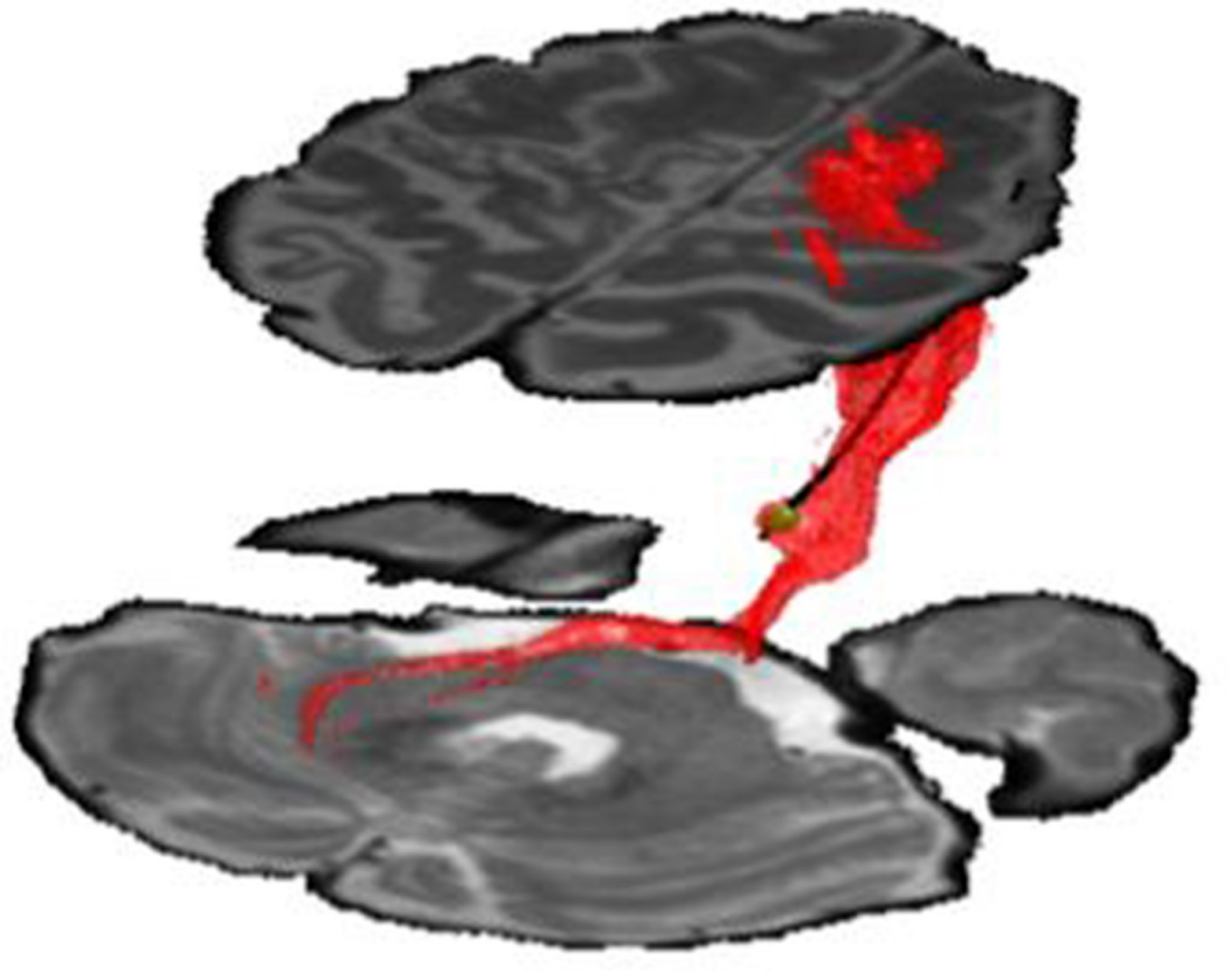
DBS electrode in anatomical location demonstrating volume of tissue activated (green volume). Fibers were generated via computational tractography (red fibers). These fibers were found to project downward to dentate nucleus of cerebellum (lower region), and upward to the premotor and M1-motor strip of the cortex (upper region). Stimulation utilized contact 1 with amplitude −1V and pulse-width 90 microseconds.

## Discussion

The prelemniscal radiations refer to a cluster of fibers that pass medial and inferior to the cell bodies of the CZi, lateral to the red nucleus, anterior to the medial lemniscus, and posterior to the sub-thalamic nucleus. The Raprl is arranged below the ventralis oralis anterior (VOA) and ventralis oralis posterior (VOP) of the thalamus. Neuroanatomical evidence suggests that the fibers of the Raprl originate from three distinct sources with different terminal regions: (1) axons project from the cerebellar nuclei, through the VIM thalamus, to the primary motor cortex (2) axons project from the internal globus pallidus, through the motor thalamus, overlap with projections from the ventrolateral thalamus (VL), and innervate the supplementary motor area (SMA), and (3) axons that project from the reticular formation. Notably, the fibers of the Raprl represent a distinct brain region separate from the cell bodies of the CZi. The Raprl has already been described as an effective target for the treatment of PD with DBS (18). Velesco, et al 2016 have hypothesized that stimulation of distinct Raprl subregions would engender improvements in the specific symptoms associated with each region’s function. For example, the cerebellar-thalamic-cortical pathway bridges the cerebellum with the primary motor cortex by way of the VIM; therefore, stimulation of these fibers may produce tremor-control that mirrors the effects seen with direct VIM stimulation. In contrast, the Raprl pathways associated with GPI-VL-SMA have been implicated specifically in the improvement of rigidity (19); these therapeutic effects are likely mediated via the decreased metabolism of the supplementary motor area engendered by DBS. The thalamic lesions observed in our patient coincide with the disruption of both the cerebellar-thalamic-cortical pathway as well as the GPI-VIM-SMA circuits; the locations of his lesions are consistent with his symptoms of tremor and rigidity. We therefore posit that the therapeutic effects of stimulation are likely mediated by these circuits. Reports of Raprl DBS for HT are rare; however, in 2004 Plaha (20) reported improvement of HT with stimulation of the neighboring CZi.

There has been ongoing debate regarding the precise mechanism of DBS, specifically whether the therapeutic benefits are the result of excitatory or inhibitory effects (21, 22). The neurophysiological consequences of DBS are thought to vary on the basis of several factors including the type of neuronal tissue being stimulated, the distance from the electrode, and the frequency of stimulation. Evidence indicates that axons are more excitable than cell bodies and that the magnitude of axonal excitability is largely contingent on the degree of axonal myelination; heavily myelinated fibers are thought to be more excitable than those that are unmyelinated (23). The therapeutic stimulation voltages reported here are lower than those described for STN or VIM in HT. Prior work has demonstrated the benefits of targeting the posterior subthalamic region, which is comprised of both the cell bodies in the CZi and fiber tracts of the Raprl (24). Rather than conventional targeting of the grey-matter nuclei of thalamus or subthalamus, our case report suggests that lower therapeutic thresholds may instead be achieved by targeting the myelinated fiber tracts of the Raprl.

## Author Contributions

VM: concept formulation, patient management and manuscript writing/editing. SH: patient selection, patient management, and manuscript writing/editing. TF: manuscript writing/editing and figures. AK: patient selection, patient management, figures and manuscript writing/editing.

## Conflict of Interest Statement

The authors declare that the research was conducted in the absence of any commercial or financial relationships that could be construed as a potential conflict of interest.

## References

[1] Holmes G On certain tremors in organic cerebral lesions. Brain (1904) 27(3):327–75. 10.1093/brain/27.3.327

[2] Krack P Deuschl G Kaps M Warnke P Schneider S Traupe H Delayed onset of "rubral tremor" 23 years after brainstem trauma. Mov Disord (1994) 9(2):240–2. 10.1002/mds.870090225 8196694

[3] Kim MC Son BC Miyagi Y Kang JK Vim thalamotomy for Holmes' tremor secondary to midbrain tumour. J Neurol Neurosurg Psychiatry (2002) 73(4):453–5. 10.1136/jnnp.73.4.453 12235320 PMC1738060

[4] Krack P Deuschl G Kaps M Warnke P Schneider S Traupe H Delayed onset of "rubral tremor" 23 years after brainstem trauma. Mov Disord (1994) 9(2):240–2. 10.1002/mds.870090225 8196694

[5] Manyam BV Uncommon forms of tremor Watts R Koller WC, Movement Disorders: Neurologic Principles and Practice. New York: McGraw-Hill (1997). p. 391–2.

[6] Gabriela R Cersosimo MG Folgar S Giugni J Calandra C Paviolo JP Holmes tremor. Etiology, associated symptoms, neuroimaging and treatment in a series of twenty cases (P2.127). Neurology (2015) 84(14 Supplement P):P2.127.

[7] Kudo M Goto S Nishikawa S Hamasaki T Soyama N Ushio Y Bilateral thalamic stimulation for Holmes' tremor caused by unilateral brainstem lesion. Mov Disord (2001) 16(1):170–4. 10.1002/1531-8257(200101)16:1<170::AID-MDS1033>3.0.CO;2-P 11215584

[8] Romanelli P Brontë-Stewart H Courtney T Heit G Possible necessity for deep brain stimulation of both the ventralis intermedius and subthalamic nuclei to resolve Holmes tremor. Case report. J Neurosurg (2003) 99(3):566–71. 10.3171/jns.2003.99.3.0566 12959446

[9] Foote KD Okun MS Ventralis intermedius plus ventralis oralis anterior and posterior deep brain stimulation for posttraumatic Holmes tremor: two leads may be better than one: technical note. Neurosurgery (2005) 56(2 Suppl):E445 10.1227/01.NEU.0000157104.87448.78 15794849

[10] Toda H Nishida N Iwasaki K Coaxial interleaved stimulation of the thalamus and subthalamus for treatment of Holmes tremor. Neurosurg Focus (2017) 42(VideoSuppl2):V1 10.3171/2017.4.FocusVid.16510 28366022

[11] Lim DA Khandhar SM Heath S Ostrem JL Ringel N Starr P Multiple target deep brain stimulation for multiple sclerosis related and poststroke Holmes' tremor. Stereotact Funct Neurosurg (2007) 85(4):144–9. 10.1159/000099072 17259750

[12] Schaltenbrand G Wahren W Atlas for stereotaxy of the human brain. Stuttgart: Thieme (1977).

[13] Butson CR Cooper SE Henderson JM Mcintyre CC Patient-specific analysis of the volume of tissue activated during deep brain stimulation. Neuroimage (2007) 34(2):661–70. 10.1016/j.neuroimage.2006.09.034 17113789 PMC1794656

[14] Chaturvedi A Luján JL Mcintyre CC Artificial neural network based characterization of the volume of tissue activated during deep brain stimulation. J Neural Eng (2013) 10(5):056023 10.1088/1741-2560/10/5/056023 24060691 PMC4115460

[15] Noecker AM Choi KS Riva-Posse P Gross RE Mayberg HS Mcintyre CC StimVision software: examples and applications in subcallosal cingulate deep brain stimulation for depression. Neuromodulation (2018) 21(2):191–6. 10.1111/ner.12625 28653482 PMC5745289

[16] Andersson JL Skare S Ashburner J How to correct susceptibility distortions in spin-echo echo-planar images: application to diffusion tensor imaging. Neuroimage (2003) 20(2):870–88. 10.1016/S1053-8119(03)00336-7 14568458

[17] Behrens TE Berg HJ Jbabdi S Rushworth MF Woolrich MW Probabilistic diffusion tractography with multiple fibre orientations: what can we gain? Neuroimage (2007) 34(1):144–55. 10.1016/j.neuroimage.2006.09.018 17070705 PMC7116582

[18] Jiménez F Velasco F Velasco M Brito F Morel C Márquez I Subthalamic prelemniscal radiation stimulation for the treatment of Parkinson's disease: electrophysiological characterization of the area. Arch Med Res (2000) 31(3):270–81. 11036178 10.1016/s0188-4409(00)00066-7

[19] Velasco F Carrillo-Ruiz JD Salcido V Castro G Soto J Velasco AL Unilateral stimulation of prelemniscal radiations for the treatment of acral symptoms of Parkinson's disease: long-term results. Neuromodulation (2016) 19(4):357–64. 10.1111/ner.12433 27075563

[20] Plaha P Patel NK Gill SS Stimulation of the subthalamic region for essential tremor. J Neurosurg (2004) 101(1):48–54. 10.3171/jns.2004.101.1.0048 15255251

[21] Vitek JL Mechanisms of deep brain stimulation: excitation or inhibition. Mov Disord (2002) 17(Suppl 3):S69–72. 10.1002/mds.10144 11948757

[22] Chiken S Nambu A Mechanism of deep brain stimulation: inhibition, excitation, or disruption? Neuroscientist (2016) 22(3):313–22. 10.1177/1073858415581986 25888630 PMC4871171

[23] Mcintyre CC Grill WM Excitation of central nervous system neurons by nonuniform electric fields. Biophys J (1999) 76(2):878–88. 10.1016/S0006-3495(99)77251-6 9929489 PMC1300089

[24] Blomstedt P Sandvik U Fytagoridis A Tisch S The posterior subthalamic area in the treatment of movement disorders: past, present, and future. Neurosurgery (2009) 64(6):1029–42. 10.1227/01.NEU.0000345643.69486.BC 19487881

